# Principles of Medicine; Comprising General Pathology and Therapeutics, and a Brief General View of Etiology, Nosology, Semeiology, Diagnosis, and Prognosis

**Published:** 1844-04

**Authors:** 


### ART. XII.

*Principles of Medicine; comprising General Pathology and Therapeutics, and a brief general view of Etiology, Nosology, Semeiology, Diagnosis, and Prognosis.* By CHARLES J. B. WILLIAMS, M.D. F.R.S., Professor of the Principles and Practice of Medicine, and of Clinical Medicine, and First Physician to the Hospital, University College, London, &c. &c.—*London*, 1843. 8vo, pp. 390.

To the philosophic inquirer into the progress of the human intellect, the revolutions of science afford a no less interesting subject of contemplation, than that presented by the revolutions of empires to the observer of the social improvement of the human race. The change of a dominant *opinion* produces as great an influence upon the actions of the mass, as the change of a *government*,—often a much greater. We do not know how much we are influenced by the principles we have espoused until we meet with others who are acting on principles exactly opposite, or find it necessary to exchange our own for some others of a very different tendency.

In no science, perhaps, have so many specious systems at different times obtained the mastery—to be in their turn overthrown by some specious or dogmatical assumption,—or have these systems exerted so great an influence over the actions and belief of those who adopted them, as in Medicine. The cause of this it is not difficult to trace. The apparent simplicity of the phenomena of disease presents great attractions to the superficial generalizer; whilst their real complexity often baffles the utmost skill of the truly philosophical inquirer. In every system, there has probably been a *modicum* of truth, however large the proportion of error with which it was mingled; and the practitioner has been naturally desirous of availing himself of any guide, by which he might simplify his treatment of the ever-varying forms of disease, without troubling himself with a minute diagnosis, or even with a careful application of his remedies to symptoms. Hence the favour which *panaceas* have met with, in all ages, among empirical practitioners *in* the profession as well as *out* of it. And hence the disinclination to the steady pursuit of pathology as a science, which those who have arrogated to themselves the distinction of *practical* men, have both manifested in their own persons, and endeavoured to impress upon others.

> "It seems quite extraordinary," says Dr. Williams, "that notwithstanding the recent rapid improvements, and comparative perfection of the contributory sciences, practical medicine should still halt in the domain of empiricism. A chief cause for the anomaly seems to be, that science and practice have been rarely pursued by the same parties. Scientific men are not and cannot be practical, because they have had no experience; and practitioners know little of science, and therefore derive little good from it. Instead of working together, these parties are at issue with each other. But it is high time to put an end to this feud. Philosophers must descend from their transcendental positions, to consider details of practice and purposes of utility. Those who would be practitioners must gain from science that knowledge and that method which renders experience instructive and useful." (Preface, p. 6.)

A better era, however, is dawning upon Medicine. The study of pathology as a science is no longer regarded by those whose opinion is most influential, as producing an incapacity for the practice of medicine; and it is now universally acknowledged that no real progress can be made except by the accumulation of appropriate materials, which shall be built up, course upon course, by the regular process of inductive generalization. This process has been going on among many of the most profound and sagacious minds of our own country during the last few years, to a degree of which few, save those who have carefully watched the indications of it, are in the least aware; and the work before us may be regarded as its first complete manifestation. We hail its appearance, therefore, not only on account of the value we are ready to attach to any production from the pen of its accomplished author, but also as the indication of a vast improvement in medical teaching, which must operate most favorably, at no distant date, on medical practice, besides giving a stimulus to many active and intelligent minds, to follow out the line of inquiry, which it has so successfully opened. The detailed examination on which we shall presently enter, will show that our anticipations are not too high; and that the work before us possesses the strongest claims on the attention of our readers.

Before proceeding to it, however, we must stop to notice the remarkable *phase* which pathological science is now assuming. We can scarcely suppose our readers ignorant of the fact, that everything now tends towards a revival of the humoral doctrines of a past age, to the overthrow or modification of many of the opinions regarding disease, which have in these later times more generally prevailed. But there is this difference between the humoral pathology of the present day and that of our ancestors: The latter was adopted as a hypothetical system, based on a very limited induction; and when once adopted, *all* phenomena were explained in accordance with it. The humoral doctrines of the present day, on the other hand, are adopted by philosophic minds, *only* so far as they are *required* by an induction from the combined results of chemical and microscopical inquiries into the constitution of the blood, and observation of the phenomena of disease; and are not entertained by any means to the exclusion of the idea that morbid actions originate, in a great variety of instances, in the solid parts of the fabric, and communicate their influence to the blood. This will be shown to be generally the case in one of the most important of all diseases—inflammation; which, on account of the peculiar interest that attaches to it at the present time, will be discussed in a separate article in our next Number.

After an excellent introduction on the need of *principles* in medicine, (which, however, we should have preferred seeing recast from the form in which it was delivered as a lecture, into one more consonant with the rest of the work,) Dr. Williams proceeds with the subject of Etiology, which occupies the first chapter of the treatise. As this part of the work is necessarily, from the fulness with which the topic has been discussed by other authors, not so original either in its plan or in its treatment as most of that which follows, we shall dismiss it with the remark that it is most admirably executed, and shows its author to be fully acquainted with all that has been ascertained on the subject, and to have carefully weighed the various theories in which the present epochs are so rife.

The introduction to the second chapter explains the plan and objects of the treatise so fully, yet concisely, that we cannot do better than transfer the whole of it to our pages.

"Disease is a change from the natural condition of the function or structure of the body; but the change is generally more or less compound, involving several elementary functions or structures; and it is obvious that we cannot obtain an accurate knowledge of the nature of disease, until we have ascertained that of its component parts. As the anatomist and physiologist examine structure and functions, by separating or analysing them into their constituent parts, before he contemplates them in combination, so should the pathologist study these constituent parts, or elements, in *disease*, before he can understand their combinations."(p. 47.)

We quite accord with Dr. Williams in the following opinion, which is put forth in a note to the preceding passage, but which seems to us of sufficient importance to be transferred to our larger type. "A neglect of this precept has greatly retarded the advancement, nay, even the formation, of pathological science. Men have begun with the very complex problems of *inflammation* and *fever* before they have made themselves acquainted with the elementary properties of textures, or even of vessels. The result has been, that the most profound reasoning and ingenious speculations have been wasted on non-entities, such as spasm of the extreme vessels, increased action of the capillaries, &c.; and even observation has been confused by the complexity of the subjects brought under it." Although there is unquestionably no *single* alteration of function, which can be compared in the frequency of its occurrence, and in the importance of its results with inflammation, yet a knowledge of its characters and effects is by no means that "one thing needful" to the physician which is commonly supposed; since many other functional alterations and structural lesions are entitled to rank with it in a *scientific* point of view, and are *collectively* of even greater *practical* importance. If our readers have any doubts on this subject, we think they will find the resolution of them in the treatise before us; our quotation from which we now resume.

"The chemist, in the examination of his subjects, finds that there are some principles or elements that cannot be analysed or divided further; these he calls ultimate or primary elements: others, again, are simple compounds, which may be analysed; but they occur so constantly, and act so singly in compounding and giving properties to complex matter, that they are called proximate principles or secondary elements. A parallel case might be shown of physical science. So should it be with physiology and pathology.* There are the healthy and diseased *primary* or *ultimate elements* of *structure*,—muscular fibre, nervous matter, vascular fibre, and the elementary tissues of membranes, glands, skin, and other parts; and there are *primary* elements, healthy and diseased, of *function* of these same structures—irritability, tonicity, nervous properties, to which may be added, because at present we cannot analyse it, the power of secretion and nutrition; and, lastly, the constituents of the blood. And there are the *secondary* or *proximate elements* of disease, composed of the preceding primary elements, but still simple in comparison with the complex conditions of disease, which they combine to produce.

* Dr. Williams adds in a note—"I have pursued this synthetic mode of teaching general pathology, in my lectures, during the last three years. I am not aware that it has been fully used by any other writer, although several (as Andral and Carswell) have partially recognized it in their divisions of the objects of morbid anatomy; and my friend Dr. Symonds has adverted to the parallel of chemistry, and actually employed the term *proximate principles of disease* in the same sense in which I use it." We believe that Dr. Symonds is fairly entitled to whatever merit may attach to the first application of this term, and to the suggestion of the train of ideas consequent upon it; his pathological introduction to Dr. Tweedie's Library of Medicine having been *published* in 1840, and probably written a year or two previously, as we have reason to know that its publication was delayed by the incomplete state of the rest of the work.—Rev.

"The following are the chief of these proximate elements: the blood-vessels and their different conditions, anemia, plethora, congestion, determination of blood, and inflammation; the nervous system, with its different functions, sensation, volition, reflected excitement, sympathy, and irritation; the secreting organs and membranes, with their relations to the vessels, the nerves, and to the purposes which the secretions serve in the animal economy; lastly, (and here we must drop physiology, for the subject is peculiar to pathology,) the elements of structural diseases, new formations, and parasitic creatures.

"These with a a few more of less importance, constitute the secondary or proximate elements of physiology and pathology; we have to consider them in relation to pathology only.

"These primary and secondary elements of disease are the especial subjects of general pathology. By the study of them we become acquainted with the materials of disease, and their relations to each other; we learn how special diseases arise, and of what they consist; how they produce their phenomena and effects, how they are to be known, distinguished, and classified. Out of such a knowledge, where it is correct, sufficient, and combined with an ample acquaintance with the properties of remedial agents, arises the rational method of curing, relieving, and preventing disease, the great ends of the art of medicine.

"I readily admit that our knowledge of these elements, these principles in pathology, is as yet too limited to be entitled to rank as a science; but I think that the attempt to describe and illustrate them will be useful, not only by making available all that is known on the subject, but also by showing what is not known, and needs investigation: these suggesting fit subjects for further research." (pp. 47-9.)

We most fully accord with Dr. Williams in these closing remarks. If no attempts are made to give to Pathology a really scientific form, it must ever remain the rude mass of heterogeneous materials, which it has until recently been. The *first* attempts must necessarily be imperfect; the *second* will be less so; and every succeeding well-directed effort will lighten the difficulty of reducing the mass of phenomena to order and arrangement. None but those who have been themselves engaged in scientific pursuits, can fully appreciate the value of these endeavours. An idea, suggested by one, in relation to a class of facts with which he is more particularly conversant, is taken up by another, and applied by him through a more extensive range. Or it becomes the indirect source, in the mind of that other, of a new mode of contemplating facts which had long been familiar to him; and thus enables him to find the hidden cord that bound them together, and to reach the mysterious principle to which they lead. We regard Dr. Williams's treatise as eminently *suggestive*; and we doubt not that its good effects will be speedily manifested; so that, by the time when a new edition is called for, many of its *lacunæ* will be filled up. These, however, are really less considerable than we had anticipated; for the materials supplied by the combination of the extensive reading and complete practical knowledge possessed by the author, and elaborated by his sagacious and philosophic mind, have rendered his modest "attempt" far more successful than we had ventured to anticipate that it could be, in the present unsettled state of opinion. We believe that one great secret of this success is his adoption of a sound and consistent physiology. His knowledge of this subject is quite *au courant* with the time; and he is fully alive to the importance of all the light which an acquaintance with the normal functions of the animal body can throw upon its morbid phenomena. We shall now proceed with our analysis.

The *first* of the primary elements, noticed by Dr. Williams, is Muscular

Irritability or Contractility; which is considered under the heads of *excess* and *deficiency*. We need not detain our readers on this section, however, as it contains no particular novelty; but we would mention that the account of the disorders of this function is based on what we believe to be the true view of its normal character,—that, namely, which regards it as an independent attribute of muscular structure.

The *second* of these elements is Tonicity; and as the operations of this property are not generally understood, we shall give our author's account of them. It may be defined as a tendency to slow, moderate contraction, not essentially terminating in relaxation; but it keeps the parts in which it resides in a certain degree of tension. This property is possessed by all muscular parts, and by some which are hardly accounted muscular. It is particularly vigorous in the lowest forms of muscular tissue, as in the fibrous coat of the arteries, and in the dartos; and in the arteries it has the function of adapting them to different degrees of fulness, whilst maintaining a certain tension favorable to equality in the motion of the blood.

"It has been asserted, that tonicity is quite distinct from irritability; and [that] although irritable fibres possess tone, tonic textures are not irritable. This is not true with regard to the arteries; for I have many times distinctly seen them slowly contract, and remain contracted, at a point to which an irritant—mechanical, chemical, or electric—has been applied. The late discovery, by Henle, of a structure distinctly muscular in arteries confirms this observation. I have proved, in like manner, the irritability of the air-tubes, which move more readily under a stimulus than the arteries; whilst that of the intestines is still higher in degree, but still inferior to that of the œsophagus and voluntary muscles, the contractions of which, on the application of a stimulus, are abrupt, and immediately followed by relaxation. So far, then, it appears, that tonicity is influenced by the same agents which excite irritability; but another agent, temperature, seems to affect them differently. Cold increases tonicity, and impairs irritability; whilst heat diminishes tonicity and increases irritability. Under the influence of cold, arteries shrink in size very remarkably;* and the muscles and other textures present a firmness and contraction which impede the quickness of motion characterizing the highest degrees of irritability. Under the influence of heat, on the other hand, although muscles are relaxed, they are more irritable, and the pulsations of the heart are more frequent.

"Cold and heat, therefore, become the best tests for tonicity; and by their means we find this property to be possessed by textures which have never been proved to be irritable; I mean the veins and the cutes, which contract by cold, and become relaxed by heat." [Regarding the non-irritability of the veins, Dr. Williams is in error; since Valentin has excited contractions in the vena cava by irritating the sympathetic nerve; and their fibrous coat, though not so distinctly muscular as that of the arteries, is yet more analogous to the non-striated muscular tissue, than to any other. The same may be said of the skin; and especially of the dartos.]

"Now this property, tonicity, is a very important one in the animal economy; its natural condition being very necessary for the preservation of health, and its modifications being concerned in causing and constituting disease. Practical men have long admitted the existence of something of this kind, without defining or localizing it; and the terms tone or atony, bracing and relaxation, tonic and relaxing remedies, become quite appropriate in connexion with this property." (pp. 53-4.)

* This fact must be familiar to every one who has noticed the difference of the pulse when a limb is cold and when it is warm. But I have seen it more forcibly illustrated by experiment. On plunging into cold water the aorta of an ass just dead, it contracted so closely as to obliterate its cavity; and it required some force to pass the little finger into it. The crimping of the flesh of fish is referrible to the same principle.

There is an *excess* of tonicity in the state commonly termed "high condition;" in which there is predisposition to disorders of a sthenic kind, but less than usual tendency to infectious disorders, and others of a depressing character; and this excess is marked by a pulse which is strong, tense, and even slow, but which is felt at the wrist synchronously with the heart's impulse. It is regarded by Dr. Williams as a chief constituent of inflammatory fever; and the remedies best suited to it are those which relax the tonic fibre and increase the secretions, together with some which seem to have a specific power of reducing it, such as antimony. We shall quote the account of defective tonicity in full; as an excellent example of the influence of sound physiological knowledge, in affording the rationale of morbid phenomena, and directing the application of remedies to their cure. We might venture to predicate, from the simple *datum* of defect of tonicity, nearly all the results which Dr. Williams enumerates as attributable to it; and to direct that treatment, which experience has shown to be most efficient. But we think that there is danger in dwelling on it too exclusively as an element of disease; since it is probably, in a great majority of instances, if not in every one, dependent upon *previous* faults of nutrition; and forms part of a complex morbid state, which renders the subject of it an easy prey to such morbific agents as malaria, &c., whose primary influence must be exerted on the blood or on the nervous system, and of whose action the body is most susceptible when *these* are in a condition below that of health. And it is further to be remembered, that, with the exception of cold, our chief tonics operate rather upon the blood and the nervous system, than upon the fibrous coat of the arteries.

"Where tonicity is defective, the muscles are flabby and incapable of continued exertion, but are sometimes too irritable, with the tremulousness of debility. The heart likewise is irritable, and often exhausts its strength in palpitation; the pulse is soft and unsteady; it may be full when slow, but it is without strength, and easily accelerated. Its most distinctive character, however, is its retardation, increasing the interval between the heart's beat and distant pulses; so that the radial pulse is often felt after the second sound of the heart is heard; the tubes being less tense, the pulse wave is slower than usual. So, too, the loose relaxed state of the vessels renders the circulation in distant parts weak, so that the extremities are cold, whilst the head may be congested. Sudden exertion or change of posture may disturb the circulation, and cause faintings or giddiness. Want of tone also in the stomach and intestines causes indigestion and costiveness, and permits them to become distended with wind and accumulating fæces. The secreting organs, irregularly supplied with blood, are also liable to disorder, being either scanty, depraved, or profuse and watery.

"It is quite obvious that a person in such a condition must be prone to various diseases. He has no resisting power against malaria, infection, or other depressing agents. If he is exposed to cold, the blood is readily driven through the weak vessels into the interior, where it causes congestion or inflammation. The weak intestines have no power to expel offending matter from them. Thus the system, in a state of atony, is liable to the action of many exciting causes of disease; besides being itself in many respects on the verge of disease.

"*Remedial measures.* The proper remedies in such a condition are tonics, or those agents that tend to increase the tone of the system, particularly of its muscular and vascular parts. We have already stated, that cold has this effect in a marked degree; and in truth cold, properly applied, is one of the best tonics which we possess. For this purpose its application should be sudden and too brief to cause depression or any of its morbid effects. The shower-bath and plunge-bath are the most effectual forms; and free sponging with cold salt water, is applicable even to weak subjects. A pure bracing air, and much exposure to it, have also useful tonic effects. There are many medicinal tonics, the most effectual of which are bark and its preparations, medicines containing iron, and the mineral acids. Generous living may be considered a part of a tonic plan, in so far as it tends to supply blood, which is the pabulum of tonicity as well as of other vital properties." (pp. 55-6.)

We think it right to take exception to this last statement; since we cannot conceive the blood to be rightly designated as the *pabulum* of a *property*. It is the material at whose expense those tissues are formed, which exhibit the several vital properties characteristic of them; and to *these* it is the *pabulum*. Were we inclined to be hypercritical, we might notice several other instances of slight inaccuracy in style, which we hope to see corrected in another edition; but we do not wish to draw off the attention of our readers from the excellencies of the production before us, to notice any but really injurious errors.

In the three succeeding sections, which have reference to the elementary Functions of the Nervous System,—sensibility, voluntary power, and reflected and sympathetic nervous influence, we do not find anything requiring special notice; these subjects having been so frequently brought under the special consideration of our readers. We shall only say that Dr. Williams is here, as elsewhere, fully alive to the pathological value of the recent improvements in physiological knowledge; and that Dr. Marshall Hall's doctrines are unreservedly adopted by him.

The sixth section treats of the Diseases of Secretion; concerning which, it is admitted by our author, that our knowledge of the properties of the secreting organs is not yet sufficient to enable us to separate these distinctly, as primary morbid elements, from disordered conditions of the blood, resulting from defect or depravation of the assimilating processes. He is, we think, quite right in the assumption, that whether the function of the secreting organs be that of *conversion*, or of mere *separation*, (the limits between which have not been hitherto well defined by physiologists,) we must attribute their selective actions to vital properties belonging to their tissues. "We are thus led to consider secretion as a peculiar property of the secernent structures, just as irritability is of muscular fibre; and as such its disorder constitutes a primary element of disease. In doing this, we avoid the hypothesis of some physiologists, who ascribe secretion to nervous influence, a notion by no means accordant with numerous facts." Nevertheless, as the nervous system is known to have a considerable influence upon the secreting process, we must anticipate morbid effects from this influence; in the same manner as we consider derangement of the normal supply of blood, as an important element in disorders of secretion. Dr. Williams justly remarks that these disorders will operate in two ways, *forwards*, on the parts to which the secretion goes, and on the function to which it is more or less subservient; —and *backwards*, on the organ, and the blood from which it is formed.

"The *forward* effects of an excessive secretion of bile depend on its stimulating properties. It irritates the intestinal tube, causing a bilious diarrhea or cholera. The symptoms of this consist in an exaggeration of those properties of the alimentary canal, which have already been described as elements of disease. Thus the irritation of the bile causes increased irritability and more rapid motion of the matter through the tube; pain from exalted sensibility; vomiting, straining, and cramps from exalted excito-motory function; profuse mucous secretion from excited secernent function." (p. 76.)

The *backward* effects of excessive secretion may be exerted either on the secreting organ or on the blood. The vital properties of the organ may be weakened by it; so that a subsequent depression follows its unusual activity, and it becomes torpid. When the excess in quantity continues for a long time the secretion is usually impaired in quality from a similar cause. The blood may have its composition much disturbed by an excess of one important secretion; which will cause an injurious predominance of the secretion that is *complementary* to it. This is especially the case in regard to the bile and urine; an excessive production of either of which, if not balanced by an increase in the other, must affect the composition of the blood injuriously.

"A clinical illustration of this position may be found in cases of bilious diarrhea or cholera. The flux of bile is either accompanied by a highly-loaded state of the urine, or by fever; in the latter case the fever does not subside until the urine becomes very copious, or deposits an abundant sediment. The most probable interpretation of this fact is, that the excessive secretion of the bile disorders the composition of the blood; so long as the kidneys rectify this disorder, by separating in greater abundance the solid contents of the urine, no fever results; but if the kidneys fail in this task, fever ensues and continues until they accomplish it; then a free secretion and copious deposit are symptomatic of the decline of the fever." [Or, we would rather say, *occasion* the decline of the fever.] (pp. 77-8)

The *backward* effects of disorder of the secreting processes on the blood are more remarkable, however, in cases of deficiency than in cases of excess. The peculiar matters of the biliary and urinary excretions are positively noxious when they accumulate in the blood beyond a certain point, and produce all the effects of poisons. Of the extent to which this accumulation may take place Dr. Williams gives a remarkable example from a case of ascites from disease of the heart, liver, and kidneys, in which Mr. Garrod obtained nearly four grains of nitrate of urea from an ounce of the peritoneal fluid, together with a considerable quantity of bright yellow solid matter, probably bilious:

"The excretions are defective in many idiopathic and symptomatic fevers, and there can be little doubt that many of the constitutional effects of these fevers are in great measure due to this important element. The positively noxious properties which excrementitious matter retained in the blood is known to possess, must be taken into account when we attempt to explain the states of constitutional irritation and depression, with perversion of functions, which fevers so generally present. The changes in the blood, manifest in some such cases by its fluidity and by petechial appearances, may also be in part referred to defective elimination of effete matter; and it is when the secreting organs recover their power, and a diarrhea occurs, or a copious discharge of highly-loaded urine, that these appearances cease. I have found purpura to be often connected with hepatic congestion and imperfect excretion of the bile, and to be most effectually removed by remedies which promote the restoration of the proper secretion." (p. 81.)

Defective secretion is of course to be restored by remedies directed towards the correction of an excess or a deficient supply of blood to the part, if the circulation through it be disturbed; and by those which have a specific influence on the secreting structure itself. The following observations on the use of the latter are of great practical value:

"But the specific stimuli of the secreting organs, if used in excess, or too long, may not only cause general weakness, but also exhaust the vital properties which they excite; and the result may be a diminution either of the secreted fluids, or of its most characteristic constituents. Hence the long or excessive use of mercury causes torpidity of the liver; that of purgatives, imperfect action of the bowels; that of diuretics, scanty urine, or albuminous or watery urine, defective in urea. These facts point out the expediency of alternating or conjoining these different agents with others calculated to improve the vital properties of the textures generally, which may often be effected by the medicines called tonic, and by regimenal means which improve and equalize the state of the circulation, and preserve the digestive and assimilative functions in the best order. In illustration of this position I may refer to the acknowledged advantage of giving bitters with or after mercurial courses; chalybeates with or after saline aperients and diuretics, when these are long used; and these additions, which alone or used at first, would check the secretion to be increased, now sustain it and render it permanent. Some medicines which are inferior in efficacy to those already named, are yet, in some instances, more eligible for chronic cases of defective secretion; because they are less exhausting, and combine some measure of tonic influence with that of increasing the secretions. As examples of this kind may be named, taraxacum, preparations of iodine, sarsaparilla, nitro-muriatic acids. Courses of these medicines are sometimes of great efficacy in keeping free the secretions, after they have been restored by more powerful means; and they likewise often improve the functions of digestion and nutrition." (p. 82.)

The morbid alterations in the circulating fluid arising from deficient secretion, are again noticed under the next head—Diseases of the Constituents of the Blood; which are treated as fully as the present state of our knowledge permits. The following introductory observations will give an idea of our author's general views, and mode of treating the subject:

"The pathological elements which we have hitherto considered are those of the vital properties of the elementary solids. We now proceed to examine the morbid changes in the blood. These, like those of the solids, may often be traced to individual elements of which the blood is composed; the changes of which must be viewed as ultimate elements of disease, and are therefore properly included in the present division. But as the blood also operates as a whole—compound, indeed, in itself, but simple in its influence on vital functions and structures—it forms a proper connecting link between *proximate* and *ultimate* elements of disease. So, also, inasmuch as it is, in some respects, an organized compound, the materials of which are changed, together with its functions, and contributes to the production of change of structure in the solids of the body, the consideration of its changes will be a proper introduction to that of alterations in the circulation, which induce changes of structure, and thus lead to structural diseases themselves.

"We have found that blood is the support of all the vital properties; and in describing their variations we have been obliged to refer frequently to differences in the supply or quality of this fluid, both as causes and as consequences of these variations. We have now to examine the properties of the blood itself; and, first, those which are most elementary or referrible to its respective constituents.

"The circulating blood consists of red particles, colourless globules, and liquor sanguinis; but as the latter is compound in function as well as in constitution, it is necessary to specify its chief constituents. We have, then, to consider:

1. The red particles
2. Fibrin and colourless globules
3. Albumen and other dissolved animal matters
4. Oil
5. Salts
6. Water

} in excess, defect, and alteration.

"Other changes affecting the entire blood are:

7. Changes by respiration.
8. ,, by secretion.
9. ,, by nutrition.
10. ,, by foreign matters.

"The average natural proportions of the chief constituents of the blood, according to Lecanu, and adopted by Andral and Gavarret as a standard, are, 127 red globules, 3 fibrin, 72 animal matter in the serum, 8 salts, 790 water." (pp. 84-5.)

Into the details of this subject we shall not follow our author, but shall content ourselves with remarking that they are treated with great judgment; and that whilst they embody, in a concise form, all, or nearly all that has been satisfactorily ascertained, they are eminently *suggestive* as to the points on which further inquiry is most needed. Take, for instance, the following paragraph on disorders of the red particles:

"Besides changes in colour, the red particles may probably be subject to alterations in their form, size, and other properties in connexion with the medium in which they are placed. It was first observed by Hewson that pure water causes them to swell, become globular, and burst; whilst saline solutions, containing more salts than serum does, make them shrink in size. These changes are now generally understood to arise from endosmosis and exosmosis; the saline matter drawing the water into or out of the little cell which constitutes the red particle. It has not been ascertained, but it is highly probable, that similar changes may take place in the living body, from circumstances which greatly alter the proportion of saline matter and water in the blood. May such change contribute to produce the serious symptoms, and even sudden death, which have ensued on drinking a large quantity of water after great exertion? Has it aught to do with the reaction and irregular excitement sometimes occurring after excessive losses of blood? Or with the symptoms of suffering which animals manifest at the instant of injecting water into their veins?" (p. 88.)

We think that Dr. Williams must have overlooked a valuable paper by Dr. G. O. Rees, in a recent number of the 'Guy's Hospital Reports,' in which the endosmotic action of the red corpuscles, and its great importance in the vital economy have received much additional illustration. In regard to the origin of the buffy coat, Dr. Williams seems inclined to the opinion—in which we accord with him—that it may be due to several different causes. The simple retardation of the coagulating process is sufficient to produce a distinct separation between the colourless and the coloured portion of the crassamentum, owing to the superior specific gravity of the red corpuscles, which have then time to sink to the bottom; "but the fibrin thus rising to the surface has neither the contraction nor the firmness of the inflammatory buff, but is gelatinous, like size, and rather resembles the sizy blood sometimes exhibited in scurvy and diabetes." Moreover, that some other cause than slowness of coagulation must generally operate, is also evident from the facts, that the buff will appear in inflammatory blood, even when it coagulates rapidly, as is sometimes the case in acute rheumatism; and that the separation takes place distinctly where gravitation can have had no influence. Dr. Williams quotes the observation of Mr. Wharton Jones,* that an increased cohesion exists between the red particles of inflammatory blood; and regards it as a valuable fact, "because it furnishes us with a microscopic test of the inflammatory condition of the blood, applicable, as Mr. W. Jones remarks, to a minute drop of blood drawn from a prick of the finger." But he does not think with Mr. W. Jones, that this increased attraction fully accounts for the tendency to separation between the red corpuscles and fibrin, by a sponge-like action of the mass of coherent particles, contracting and squeezing out the fibrin from between them, before it

* British and Foreign Medical Review, October, 1842.

coagulates; "for," Dr. Williams remarks, "I do not consider this comparison a just one; for, so far as I have seen, the cohesion of the red corpuscles is not in an entire mass, but only in separate piles or rouleaux; these would facilitate the separation, not only by contractile aggregation, but also by sinking through the liquid fibrin more quickly than separate particles would; just as bits of chalk fall to the bottom of water, instead of remaining long suspended, as they would do in fine powder." That the increased tendency to aggregation among the particles of the fibrin has at least as much to do with the production of the inflammatory buff as the attraction of the corpuscles, appears to us fully proved by the fact, that the colourless coagulum is much firmer than usual, its fibrous structure much more evident under the microscope, and its subsequent contractions much more powerful. The relations of fibrin to the normal and morbid processes of nutrition, are concisely set forth in the following paragraph; the general views contained in which are expanded in the subsequent part of the treatise:

"Fibrin, or the buffy coat of the blood, is also the material of which new membranes and cicatrices are formed, constituting the *coagulable lymph,* which is the plasma or basis of the constructive or reparative process. But in its capacity for this process fibrin exhibits some varieties. The plasma with which old textures are nourished and new ones formed, is *euplastic* in a healthy state, having a capacity of life: and may become organized in a high degree, as in false membranes resulting from acute inflammation in a healthy subject. But in many instances this capacity is degraded, and the nutritive material is *caco-plastic,* susceptible of only a low degree of organization, as in the indurations resulting from low or chronic inflammation, fibro-cartilage, cirrhoses, gray tubercle, &c.; or it is *aplastic,* not organizable at all, as in pus, curdy matter, yellow tubercle, &c. It is a point of great importance that the quantity of fibrin in the blood and the facility with which it may be effused are by no means in proportion to its plasticity, or capacity to become organized; thus it is abundant in the blood, and freely effused in the inflammations, of scrofulous and tuberculous subjects, although the products of these inflammations and of nutrition, are commonly caco-plastic or aplastic. It is interesting to observe that in these cases also the red particles are defective in number." (p. 98.)

We regard the question of the means of reducing the quantity and diminishing the plasticity of the fibrin of the blood, when these are in excess, as one of the most important problems in medicine. It has been clearly shown that general bloodletting possesses but little direct control over the process by which fibrin is elaborated; for even large detractions, in a case of acute inflammation, scarcely diminish the proportion which the blood contains in any perceptible degree. The following points, therefore, are well worthy of consideration:

"It would probably be found that purgatives and other remedies which increase much the more solid secretions, diminish the fibrin. A similar property has been ascribed to mercury, to alkaline salts, to iodine, and to antimony. I know of no positive facts in support of this notion, but it is favoured by some analogies, and seems well worthy of experimental investigation. The operation of salts and alkalies in this way was probably suggested by their property of dissolving fibrin out of the body."* (pp. 99-100.)

* "My friend Mr. Blake has made many experiments of injecting various saline and other fluids into the veins; and he has furnished me with a summary of their effects on the blood, as found after death. The blood was found coagulated after the injection of the following matters: Liquor potassæ (firmly); carbonate of potass (firmly); nitrate of potass (firmly, blood scarlet); nitrate of soda; nitrate of ammonia; nitrate of lime;

The following is a suggestion of great practical importance :

"Bloodletting and other general antiphlogistic remedies, if they do not remove local inflammation, may render its products more injurious, by lowering their plasticity, and approximating them to tuberculous and other aplastic deposits. Thus chronic inflammation continuing after the full application of the antiphlogistic treatment, almost surely tends to produce degenerated changes of structure, over which remedial art has little power. In connexion with this subject, therefore, we see how desirable it is that inflammations should be removed before they become chronic; and when there is a risk of their becoming so, it should be an indication to improve the condition of the blood by a tonic and nutritive plan, at the same time that local antiphlogistic measures may be necessary for the lingering inflammation." (pp. 218-19.)

The combination of local depletion with a general tonic and even stimulating plan, though ridiculed by the routine practitioner—who has no idea of any medium between blue pills, black draught, and starvation, on the one hand, and iron, quinine, and generous diet on the other—is one which many of our highest medical authorities have united in recommending in cases of this kind; and we think the rationale of its success offered by Dr. Williams is very satisfactory. "A similar tonic treatment," he adds, "is still more indicated in scrofulous, chlorotic, and other cachectic states, in which the fibrin, though less abundant than in inflammation, is yet copious in proportion to the scanty red particles. Hence the tendency to the deposit of imperfect fibrin, even independently of inflammation." We are inclined to regard the normal proportion of *red* particles as a most important condition of the due elaboration of the fibrin, whether or not they be the real instruments of this elaboration. That the respiratory process to which they minister has a close connexion with the generation of the fibrin, seems evident from the more perfect character of the fibrin of arterial blood, which cannot be reconverted into albumen in the same manner as that of venous blood; and we would suggest it as an interesting subject of inquiry, whether the fibrin of tuberculous subjects does not resemble venous rather than arterial fibrin. The distinction between them, as stated by Scherer, is this: Venous fibrin (as first made known by M. Denis) may be entirely dissolved in a solution of nitrate of potash; and this solution, coagulable by heat, greatly resembles a solution of albumen. But arterial fibrin, or even venous fibrin long exposed to the air, is not thus soluble; and when a solution of venous fibrin in nitre is exposed to the air, a fine flocculent precipitate, having the characters of arterial fibrin, is gradually formed at the surface, and falls to the bottom. The recent experiments of Mulder seem to indicate that the protein of these two forms of fibrin may be in different states of oxidation.

Of the Changes of the Blood by Respiration, which are discussed in section XIII, we need say little; since their usual effects are familiarly nitrate of baryta; chloride of calcium; chloride of barium; chloride of strontium; sulphate of magnesia; sulphate of copper; acetate of lead; arsenite of potass; nitric acid (strongly); narcotin (firmly); tobacco; strychnia (moderately); conium; hydrocyanic acid; euphorbium; and water in quantity. The blood was not coagulated, or imperfectly so, after injection of caustic soda, carbonate of soda, sulphate of soda, ammonia, nitrate of silver, sulphate of zinc, sulphate of iron, phosphoric acid, arsenic acid, arsenious acid, oxalic acid, infusion of galls, of digitalis, alloxan. Some of these results are different from what might have been expected; instance the decided coagulation with potass and its salts, especially nitre, and the fluidity with nitrate of silver, sulphate of zinc, infusion of nutgalls, which have been commonly supposed to possess a coagulating property." (pp. 100.)

known, and it is doubtful how far they can be disordered in any other way than by *deficiency*. On the idea of Liebig, that excessive oxygenation of the blood may be a cause of disease, Dr. Williams makes the following judicious remark:

"It seems to me that Professor Liebig has given too mechanical a view of the change of the blood in respiration. He appears to consider the increased arterialization of the blood, during exercise and on exposure to cold, to be a necessary consequence of the greater amount of air inhaled, in one case by accelerated movement of the chest, in the other by the greater density of the cold air. But if the extent of the changes wrought by respiration were in exact proportion to the quantity of oxygen received into the lungs, how easy would it be to increase them (and thereby animal heat also,) by voluntarily augmenting the respiratory movements. I cannot but think that the proportion of oxygen absorbed, and of carbonic acid formed [given off?] depends more on the condition of the blood brought to the lungs; and that the respiratory movements are regulated by this. Thus the increased oxygenation of the blood is a consequence, not a cause, of previous changes previously wrought in the blood itself." (p. 108.)

In this last sentence there is an error, of which a captious critic might take advantage; for how can anything be a *cause* of *previous* changes? The meaning, however, is quite obvious. Dr. Williams regards the changes taking place in the lungs as only the *exponents*, so to speak, of the changes taking place in the systemic capillaries; the former having for their object to balance, by alterations of a converse nature, the effects produced by the latter. In this we fully agree with him; being satisfied that any attempts to account for phthisis, for example, upon the supposition of an increased oxygenation of the blood, *causing* more rapid disintegration of the tissues, will lead us away from the right line of inquiry, —the state of the nutritive processes, on which the perfect formation of the tissues, and its greater or less tendency to disintegration are dependent.

We think that the section on the Changes of the Blood by Secretion, might have been somewhat extended with advantage, by a fuller notice of various recent inquiries, which rightly fall under this head. Thus, Dr. G. O. Rees's paper, already adverted to, furnishes some views of great importance in regard to the influence of the state of the blood in producing albuminuria; and their action of the disease, when established, upon the condition of the blood. His idea that the albuminuria which occurs after scarlatina, is due to the complete obstruction to the perspiration, which the state of the skin produces—whence are occasioned increased determination to the kidneys, and dilution of the liquor sanguinis by the retention of the watery part of the blood—strikes us as extremely ingenious; accounting, as they do, for the absence of the serious results which we should naturally expect to follow such an indication.

We have only room for one extract from this section:

"The *perspiratory secretion* contains lactic acid and lactates of soda and ammonia, which probably proceed from the transformation or decay of the textures, particularly the muscular. Hence these products abound during great muscular exertion; and when perspiration is checked by extreme cold, they may be retained in the blood, causing rheumatism, urinary disorders, or various cutaneous diseases. The very serious effects sometimes resulting from sudden cold on the perspiring body may be partly owing to the same cause, as well as to the disorder produced in the circulation. Rheumatism is especially liable to occur as an effect of cold, where the body is fatigued with much muscular exertion; and I have frequently observed that the rheumatism chiefly affects the limbs which have been most exercised. Where the skin fails to excrete, an increased task is thrown on the kidneys, whence may result various diseases of these organs; and if these organs fail in the task, the lactic acid accumulates in the blood, and, probably acting as a ferment, causes the formation of more, and of the kindred products, lithic acid and its compounds: these, in inflammatory subjects, excite rheumatic fever and, in more torpid frames, various rheumatic or gouty affections. All these cases are frequently remarkable for the acid character of the cutaneous and renal excretions.

"The remedies for rheumatism, therefore, should not be merely antiphlogistic, but also of a kind calculated to eliminate the morbid matter from the blood. In slight cases sudorifics may suffice, but in others the kidneys and liver should also be excited to assist in the process of elimination; and various combinations of colchicum and alkalies with mercury, opium, and iodide of potassium will generally effect this very satisfactorily."* (pp. 115-16.)

We would add to this a strong recommendation in favour of the hot-air bath, which we have known, since our former notice of its effects, (vol. XVI, p. 471,) to produce most beneficial results.

In the following section, on the Changes in the Blood from the Transformation of Chyle and of the Textures, Dr. Williams has confined himself to the two remarkable states on which chemistry has recently thrown so much light—namely, *gout* and other *lithic acid diseases*, and *diabetes.* His opinions on these are quite in accordance with the views we have ourselves expressed in former articles; and he considers, as we do, that there is a danger of pushing merely chemical explanations further than they are borne out by the actual phenomena of disease. That he has done wisely in abstaining from introducing into this section many of the hypothetic views in which the last two or three years have been so fruitful, we are well assured; knowing their seductive influence on the mind of the student.

The last division of the Primary Elements of Disease, includes the changed properties of the blood, from the presence of foreign matters; a topic of the most extensive kind. Too little is yet certainly known, however, respecting the mode in which these foreign matters are introduced into the blood, and the effects which they produce upon its character, to enable much more to be stated than the following:

"The blood is probably the chief seat of the morbid poisons which excite various contagious, epidemic, and endemic diseases. Probably, too, it is the hotbed in which some of them are propagated; and it is through changes in its composition, that many of the destructive effects of these poisons are produced. We have already noticed some of these changes under former heads. It will suffice in this place to mention a few examples, in which morbid poisons have been traced to the blood........ There is good reason to suppose that purulent matter, and the germs of carcinoma and other forms of malignant disease, are spread through the system through the medium of the blood. The tendency to symmetrical arrangement which cutaneous eruptions, nodosities of the joints, paralysis from lead, and some other local affections exhibit, has been adduced, by Dr. W. Budd and others, as an instance of effects produced through the medium of the blood,—the symmetrical distribution of this fluid on the opposite halves of the body leading to like results in corresponding parts." (pp. 121-2.)

* "The advantages of this due regard to all the elements of disease in the treatment of rheumatism may be shown by the fact, that, with few exceptions, I have found that three or four days suffice to remove the fever and pain in the severest forms of acute rheumatism." (p. 115.)

The following judicious observations conclude this division of the work. It is interesting to observe, how completely the science of the pathologist and the experience of the practitioner are now agreed in the propriety of drawing off the "peccant humours" from the circulating fluids, by remedies which act through the natural excreting processes. We would suggest as an addition to Dr. Williams's remarks, whether the practitioner should not choose as his special "emunctory," in each form of disease, that excreting organ, which appears to have a natural tendency to eliminate the particular kind of morbid matter from the blood; thus rather aiding the efforts of Nature, than attempting to set up an altogether new kind of action in the body;

"In the treatment of this element of disease, foreign morbific matters in the blood, the two indications which present themselves are: 1, to counteract the injurious operation of these matters; and 2, to expel them from the system. The first of these indications is followed, when we give stimulants to overcome the depressing influence of adynamic fevers and other sedative poisons; and when opium and other narcotics are administered where irritation prevails. We do not possess chemical antidotes which can act on the foreign matter in the blood, without injuring the blood itself. The other indication is more generally pursued, although little recognized by practitioners, to expel the offending matter from the system. The excretory organs, especially the kidneys and alimentary canal, are the natural emunctories through which foreign and offending matters are expelled from the blood; and hence the utility of alterative aperients and diuretics in the treatment of fever and other diseases connected with poison or injurious matter in the blood. Orfila found that the pernicious effects of small repeated doses of arsenic in animals might be averted by giving at the same time a diuretic medicine. Let us bear in mind how often fevers and other serious ailments seem to be carried off by spontaneous diarrhœa, diuresis, or perspiration; and, perhaps, sometimes by these discharges artificially excited. Nor should a converse fact be overlooked, that persons affected with disease of the kidneys (cacoplastic degeneration) which impairs its excernent function, are peculiarly liable to contract infectious diseases, and to suffer from their effects." (p. 122.)

We now pass on to our author's next division; which includes the Secondary or Proximate Elements of Disease, consisting of two or more Primary Elements. Of these *proximate elements*, the class most generally studied comprehends those which relate to the circulation of the blood; and here we find at least three of the primary elements already noticed, to be concerned: the blood and its constituents, the muscular action of the heart by which it is impelled, and the tonicity of the arteries which regulates its distribution. To these we might add, the functional state of the organs to which it is distributed. The first subject considered is *General Anemia;* a state with which our readers must be very familiar. One of its most remarkable features is the functional excitement of the nervous system, which so frequently manifests itself, and which so strongly resembles that occurring under an entirely opposite condition of the general system. No explanation of this apparent anomaly has been proposed; but Dr. Williams suggests one, which we think well deserving of consideration. When the mass of the blood is reduced in quantity, the blood-vessels of the body at large contract in similar proportion, their tonicity adapting them to the amount of their contents. But the blood-vessels within the skull and spinal canal cannot contract with the same facility; for not being exposed to atmospheric pressure, and some of them being fixed in bony canals, they do not shrink as the blood becomes reduced, and therefore they retain more than their proper share of the circulating fluid. This statement is not invalidated by recent experiments of Dr. G. Burrows (Medical Gazette, April, 1843.) His experiments and observations very satisfactorily demonstrate the absurdity of the notions, founded on Dr. Kellie's paper, that the quantity of blood in the head is always the same; but it remains clear that the circulation within the head and spinal canal, especially in man, is affected by losses of blood, differently from the circulation in other parts. Now this disproportionate amount of blood in the nervous centres produces different effects, according to the degree in which the heart's propulsive power reaches it. Under the influence of palpitation, fever, or other kind of excitement, the brain and spinal cord receive through their uncontracted vessels an unusual share of the force of the heart; an erethism of some one or other of the functions of these nervous centres is the consequence; and pain, spasm, sensorial disturbance, or sympathetic irritations of some kind or other occur. There seems to be the same *disproportion* between the circulation in the nervous centres and that of other parts, in the state of anemia, as there is when the total amount of blood is normal, but an excessive determination of it exists towards these parts. That this disproportion should be greatly increased, by a very slight excitement of the heart's action, will be evident from the consideration that when the arteries are full and tense, they oppose their fulness and tension to each contraction of the heart; which resistance reduces the strength of each pulse in the vicinity of the heart, although it contributes to propagate it to a distance; but when the arteries are loose and empty, the heart squirts the blood into them in an unresisted jet, the force of which is strong near the heart, but extends not to distant arteries. This idea is further applied by Dr. W. to explain the increased arterial pulsation of other parts *near* the heart, in anemic subjects,—giving rise to the painful throbbing often complained of in the throat, chest, and epigastrium,—even when there is little pulse in distant arteries, and the extremities are cold.

But such a state of circulation will not only produce nervous excitement when the heart's action is but slightly increased, but undue depression of the nervous functions when it is feeble. For it may be then inadequate to propel the blood accumulated in the vessels of the brain; the blood therefore stagnates, and may cause some of the symptoms of congestion in that organ, the accumulation being greatest in the veins and sinuses, and the motion of the fluid not being at all proportionate to its quantity. Such congestion may be only temporary, and may lead to no serious results; but Dr. Williams thinks that in some cases a coagulation of blood in the sinuses, and a consequent permanent obstruction to the passage of the blood through the vein, may be the result. He states that he has met with at least three cases of the following description.

"A young female becomes anemic, and after exhibiting various symptoms of feeble general circulation, with headach, drowsiness, and impaired sensorial functions, suddenly becomes worse; passes into a state of stupor, with dilated pupils, sometimes varied by slight manifestations of delirium, throbbing of the carotids, and partial heat of the head, and dies comatose. On opening the head a small quantity of serum is found under the arachnoid and in the ventricles, sometimes with a little lymph (in one case there was none.) The vascularity of the membranes is remarkable, but the vessels most distended are the veins; and in the larger of these, and in the longitudinal sinus, there is a firm coagulum. In parts, especially at the torcular hierophili, this coagulum blocks up the whole sinus, and exhibits a separation of fibrin, portions of which are softened down into that purilaginous matter which was long mistaken for pus, but which Mr. Gulliver has shown to be a physical change of the fibrin which mere stagnation may effect. These have been taken for cases of meningitis. No doubt inflammation does supervene in them occasionally; but in two cases that have fallen under my notice, there was no adhesion of the arachnoid nor deposit upon it, nor any other unequivocal mark of inflammatory action; yet the fibrinous and bloody concretions in the veins and sinuses were the most remarkable for their size and firmness.

"It appears to me most probable that these affections originate in the encephalic congestion connected with anemia. Fibrinous concretions form on the transverse bands of the sinuses, and increase till they considerably obstruct the passage of the blood: hence the impaired state of the cerebral functions, amounting at last to coma. Reaction may take place with determination of blood, and even inflammation; and these may cause the symptoms of partial excitement that sometimes exhibit themselves; but neither during life, nor on examination after death, are the proofs of excitement so prominent as those of obstruction and interruption to the cerebral functions. It must be remembered that in anemia the fibrin of the blood is not diminished in the proportion of the other animal contents; and it has a greater tendency to coagulate than in healthy blood." (pp. 127-8.)

We have ourselves witnessed a case of this kind, in which, to the surprise of the medical attendant, and the grief of the surrounding friends, a sudden transition took place, from a state which was considered one of mere functional derangement, and expected to be easily subdued in time by well-directed treatment, to one of fatal oppression; no post-mortem examination having been permitted, however, we cannot speak as to the immediate cause of death; but Dr. Williams's rationale of it seems to be very consistent with the symptoms, and with the general state of the system. A representation of the morbid appearances presented by a case, which appears to have been similar in its results, is given by Cruveilhier; but the case was considered by him, as Dr. Williams thinks, without sufficient reason, to be one of cerebral phlebitis. We think it worthy of consideration, however, whether some degree of this affection may not participate in the result; since phlebitis is a disease of frequent occurrence in subjects of an anemic character.

We are compelled to pass on rapidly to *Hyperemia*, under which general term Dr. Williams includes several distinct affections; his arrangement of which, indicative of his idea of their mutual relations, we shall extract:

"Too much blood in the system, or in a part, is a most frequent element of disease. It implies an undue distension of the vessels which contain it; and a modification of the properties of these, and of the heart which propels it, is almost constantly a concomitant of this morbid condition. The chief vital properties of the heart and vessels are irritability and tonicity: excess and defect of these form most important elements, which modify the effects of excess of blood; and thus is suggested synthetically a division (long recognized as most important in practice) into active or sthenic, and passive or asthenic hyperemia, which distinction is applicable to both the general and the partial excess of blood. Another variety of hyperemia may be distinguished by an altered or perverted action of the vessels which is chiefly applicable to the affection in a part, and includes that singular and complex condition—inflammation. A view of these important proximate elements of disease is given in the following table. It is not meant that the diseased conditions here specified are always separate, or that they consist merely of the elements here stated; but these are their most distinguished parts, and most important in regard to treatment.

| | | | Results. |
|---|---|---|---|
| Hyperemia: excess of blood. | General, *Plethora* | with motion increased = *Sthenic*. | Hemorrhage, |
| | | with motion diminished = *Asthenic* | |
| | Local. | With motion diminished = Congestion | Flux, |
| | | " increased = Determination of blood | Dropsy. |
| | | " partly increased / " partly diminished = Inflammation. | (p. 132.) |

We are disposed to question whether the difference between the *sthenic* and *asthenic* forms of plethora consists *alone* in the different proportions of the strength and irritability of the moving fibre, as Dr. Williams seems to think. (p. 134.) It appears to us to depend at least as much upon the state of the blood, in regard to the proportion between its different constituents; and upon the relative activity of the formative process concerned in nutrition and secretion, which results from the degree of the vitality of the solid tissues, and which seems to us to be rather the cause than the consequence of the variations in the degree of contractility and tonicity in the heart and arteries. In the *sthenic* form of plethora, we believe the fibrin or plastic element of the blood to be in abundance, as well as the red corpuscles, and the formative process to be active. (This, indeed, is admitted by Dr. Williams, but not in a way which would lead the student to regard it as the essential character of the condition.) Consequently, there is at least as much tendency to inflammation as to hemorrhage; the inflammatory action being liable to occur whenever a lowering of the vitality of a part takes place, whereby the consumption of the fibrin will be diminished. If hemorrhage occurs, it will be in its active form, dependent rather upon a bursting of the vessels by increased pressure from within, than upon the weakening of their walls, which is the character of a passive hemorrhage. Now in asthenic plethora we find both the causes and consequences to be such as indicate a decided deficiency of the fibrin in the blood. It especially affects those weakened by age, excesses, or previous disease; and it tends to produce congestions and passive hemorrhages, fluxes or dropsies, with a general torpid state of the functions, occasionally interrupted by excitement. In this state, as it appears to us, the amount of fibrin is probably low in proportion to that of the corpuscles, and there is less tendency to inflammatory diseases. Hence, although bleeding may relieve this form of plethora, it does not produce the same health-restoring action as in the preceding case; for the deficiency of the fibrin, and the low condition of the formative processes, may require a tonic and even stimulating plan, with nourishing diet, at the very time that blood is being drawn, and the secretions being increased by evacuants, to remove the morbid matters that may have accumulated in the blood, in consequence of their previous scantiness. This is the plan of treatment indicated by Dr. Williams himself, as likely to restore the " lost tone to the over-distended vessels;" but we doubt if it effects this in any other way than by increasing the production of well-elaborated fibrin, whilst the red corpuscles are brought down to their normal standard by bloodletting; and thus restoring that normal proportion of the chief constituents of the blood, which is essential to the regularity of the circulation, and to the healthy nutrition of the solid tissues.

We think it right to state our opinion, that, on all points connected with the movement of the blood, there is a deficiency in Dr. Williams's reasoning, arising out of his non-admission of any power, independent of the action of the heart and arteries, operating upon the blood during its motion through the capillaries, and either accelerating or retarding its passage. The chief arguments in favour of the existence of such a power we have already recapitulated on more than one occasion; and our limited space prevents us from here entering upon the question in full. We think it right to state, however, that our conviction of the existence of such a power has been strengthened, rather than impaired, by further consideration; and that the denial of it can only be sustained by a very limited survey of the facts bearing upon the question,—the most comprehensive view of these facts being that which affords the strongest support to the position we have assumed. This position is,—that in the lowest tribes of animals, as in plants, a regular circulation of the nutritive fluid is maintained, without the aid of mechanical propulsion, by forces generated during its movement, and dependent upon the changes to which it is subservient; that, as we ascend the animal scale, we find the peripheral force gradually giving place to a central one, created by a muscular organ of impulsion; but that, even in the highest animals, in which this concentration has proceeded the furthest, the peripheral force still regulates the distribution of the blood, in such a manner that by its increase in a part it shall cause a local determination of blood, whilst by its diminution it shall occasion a partial or complete stagnation. This view is not in the least inconsistent with the doctrine, that the heart's action, in the higher animals, is adequate to force blood from arteries to veins without any other aid; in support of which doctrine the following experiment by Dr. Sharpey is cited by our author:

"A syringe, with a hæmadynamometer to show the amount of pressure used, was adapted to the aorta of a recently-dead animal, the vena cava being divided. Warm water was then injected, and with a force that raised the mercury in the hæmadynamometer only three inches, the water passed through the capillaries and out of the vena cava. When the pressure was increased, so as to raise the mercury six inches, the flow was very free; and on adapting another hæmadynamometer to the vein, the pressure in this was found to rise as high as three inches. The pressure thus used in the arteries (six inches of mercury) was not greater than the natural pressure in the arteries of the living animal; and the pressure transmitted to the veins (three inches of mercury) was greater than that in the veins of a living animal." (p. 143, note.)

It is not proved by this experiment, however, that the circulation can be maintained by such an amount of pressure with its normal rapidity, which is a most important element of consideration. Nor does any theory of the circulation, which rests upon the heart's action alone as the moving power, explain why the blood moves through the vessels much more rapidly (as from the experiments of Hering and Blake it would seem to do) than the force-pump action of the heart could account for. And we take exception to inferences drawn from any experiments of this kind, made with water upon the vessels of a dead subject, as not affording results which can be fairly brought into comparison with those afforded by observation of the circulation of blood in the living vessels under a variety of conditions. For example, it has been found easy to make fluid pass from the arteries to the veins of a gangrenous limb after its removal from the body, although no blood would pass during life; and as no mechanical impediment to its passage could be detected, and the heart's action was unimpared, it seems evident that something was wanting to complete the moving power required. Such a case is very parallel to that of asphyxia, in which a similar stagnation results, not from the death of the pulmonary tissue, but from suspension of the changes involved in the aeration of the blood. Perhaps the most striking proof that some such peripheral aid must operate in maintaining the circulation of blood, is derived from the case of acardiac fœtuses. For even if we receive the ordinary explanation of their circulation, that it is carried on by the heart of the twin perfect fœtus, always (or almost always) associated with the monster, we cannot help admitting the existence of some additional power; since the heart is not found more powerful than usual, although it has double duty to perform, and can therefore only exert half its usual impulsive force upon the blood of each individual; whilst even this half must be exerted at a great hydraulic disadvantage upon the blood in the vessels of the acardiac fœtus. But our position is still further strengthened by the fact, of which we believe that there is demonstrative proof, that the circulation in the acardiac fœtus was, in one case at least, independent of that of the perfect twin. The case we allude to is one of which an account was given by Dr. Houston, at the meeting of the British Association in 1836; of which account a summary will be found in our Second volume. A different explanation has lately been proposed by Dr. Marshall Hall ('London and Edinburgh Monthly Medical Journal,' vol. vi, p. 541 ;) but at the last meeting of the British Association, Dr. Houston again brought forward the subject, and completely rebutted Dr. Marshall Hall's arguments (in our opinion at least,) proving that the influence which he imagined to be in operation *could* not have been propagated from one fœtus to the other, consistently with the simplest principles of hydraulics, and with the facts ascertained by anatomical examination. But on this question our readers have now the opportunity of forming their own judgment, by examining Dr. Houston's reply for themselves; it being now published in the 'Dublin Journal of Medical Science' (January, 1844.) It may still be objected that the case, being that of a *monster*, no inferences can rightly be founded upon it in regard to the circulation in perfect animals of the same class. To which we reply, that we should consider it absurd to argue from such a case, that the heart is not generally necessary for the sustenance of the circulation; but that we regard it as satisfactorily proving that there *is* a supplementary power, arising in some way or other out of the passage of blood through the living tissues, and the actions to which it is there subservient; which power may, under the peculiar circumstances of the case, be so increased or exaggerated as to perform the whole, or nearly the whole, of the duty.

We have no wish to be thought to dogmatize on this subject; but when we remind our readers that the opinions we entertain were strongly urged by Haller, and have been supported in recent times by Professors Alison and Graves, and by many of their most intelligent pupils, we think they will agree with us that they are not to be hastily set aside by experiments such as the foregoing. We may fearlessly ask whether the condition of the dead body, with the syringe and hæmadynamometer fitted to its vessels, was not more *monstrous,*—that is, more aberrant from that normal state it was intended to illustrate,—than the condition of the acardiac fœtus, with living blood circulating through its living vessels, and ministering to its actions of nutrition, secretion, &c.

We should not do justice to Dr. Williams, however, were we not to state, that whilst neglecting what we believe to be one essential element of the morbid conditions in question, he displays peculiar ingenuity in working out the results of physical causes affecting the movement of the blood; and proves that they may produce effects much greater than could have been anticipated. Thus he states that atony of the small vessels is a cause of congestion, not only by making them yield and become distended by the accumulation of blood, but also by rendering them unfit to transmit the force of the current in its proper direction, on account of their tortuosity and want of elasticity; and he illustrates this position by the following experiments, the results of which are very striking:

"To one of Read's enema syringes was adapted a tube with two arms; to one arm was fitted a brass tube two feet long having several right angles in its course; to the other arm was tied a portion of rabbit's intestine, four feet long, and of caliber (when distended with water) double that of the brass tube. The intestine was placed in curves and coils, avoiding angles and crossings, which might obliterate the canal. The discharging end of both tubes was raised to the same height; that of the intestine being kept open by a short tube of metal. The tubes were then both filled by successive strokes of the piston; and when they both began to discharge, the quantity received from each, in a given number of strokes, was ascertained. Without giving the details, it may be stated that the small metal tube discharged from two to five times the quantity discharged by the larger but membranous tube; the difference being greatest when the strokes of the piston were most forcible and sudden, by which the intestine, although much swelled at its syringe end at each stroke, conveyed comparatively little water. The difference was further increased by raising the discharging ends higher; and when both ends were raised to the height of eight or ten inches, the gut ceased to discharge, each stroke only moving the column of water in it, but this subsiding again, without rising high enough to overflow. On increasing the force of the stroke, the part of the intestine nearest to the syringe burst. The experiment was repeated in various ways, of which I will mention one, with a metal tube two feet eight inches long, and a bore three eighths of an inch, and a portion of dog's intestine of the same length, but when distended, of double the diameter. The metal tube yielded three times more liquid than the intestine." (p. 145.)

We quite agree with Dr. Williams in thinking that, although the experiments exaggerate the difference between healthy and relaxed or congested vessels, yet they really prove that the increased tortuosity and number of vessels in a congested part, the greater mass of their contents, and the atonic flaccidity of their coats, do truly form additional obstacles to the passage of blood through them, although the amount of these obstacles will vary according to the state of the connected circulation.

The loss or neutralization of the propulsive force, by *misdirection*,—in consequence of its being partly expended in distending or dilating the nearer portion, whilst a sufficiency does not remain for the onward propulsion of the blood,—is a principle which seems to us of very high importance, in explaining many anomalies of unequal circulation. But still, we think, there is another influence which contributes to the stagnation of blood in the capillary vessels, and which is frequently its primary cause; we refer to the cessation or diminution of the actions which should take place between the blood and the surrounding tissues, during its passage through the capillary vessels. Of this influence, we consider the condition of asphyxia, in which the actions that ought to take place in the pulmonary capillaries are entirely suspended, as a *glaring instance;* and Dr. Williams does not altogether deny its operation, although he seems to think, that further inquiries will reduce it to physical causes. We regard asphyxia, however, not as an insulated case, but only as one instance, more striking than most others, of the general fact, that depression of the proper function of an organ,—whether that function be nutrition or secretion,—will cause stagnation of blood in its capillaries, or congestion. Even this seems partially admitted by Dr. Williams, as the following passage shows: "Congestion occurs in various organs and surfaces, when their proper secretions are arrested, or suddenly diminished. It is difficult to determine whether the congestion is the cause or the effect of the defective secretion in the first instance; and very probably the relation is mutual: at least this is the most convenient view to take of the matter, for practical purposes. Thus, means which increase the secretion will often remove the congestion; and those which relieve the congestion generally restore the secretion." (p. 297.) We have no doubt that Dr. Williams is right in asserting that such a mutual dependence exists; since experiment shows, on the one hand, that obstruction to the circulation through a part, artificially induced, produces diminution in its natural secretion, whilst often leading to an abnormal discharge or flux; and, on the other hand, the intelligent practitioner is familiar with the fact, that there are forms of congestion which no withdrawal of blood from the part can cure, but which are immediately relieved when the natural secretion is re-established,—just as the congestion of the pulmonary arteries, right side of the heart, and venous system, in asphyxia, is relieved as soon as oxygen can be made to act on the blood in the pulmonary capillaries. We think the distinction a more important one in practice than Dr. Williams seems to regard it; and should be inclined, whenever we fail in detecting any *extraneous* cause for the congestion,—such as obstruction in the lungs, or an imperfectly-acting heart, in a case of congestion of the liver,—to ascribe it to causes having their seat in the part itself, and to treat it accordingly. Doubtless, in almost every case of congestion, a share of the effect will be produced by the *diminished tonicity* of the vessels of the part, in the manner so well illustrated by Dr. Williams; but this we should often regard as rather a *consequence* of the depression of its general vitality, than as its *cause.* Thus it is in states of general debility, that we find gravitation or any other local causes most efficient in producing congestions.

There are many points of great interest in this chapter, which we should gladly stop to notice, did our limits permit. Dr. Williams is inclined to assign to congestion a larger share in the production of some abnormal conditions, than most pathologists will be ready to admit; but we think he is probably right in so doing. Thus he believes that not only *serous* or *albuminous*, but even *fibrinous* effusions may result from congestion; the former occurring when the blood is poor, so that its watery parts easily pass from the congested vessels, even without much distension; whilst the latter result from the greater richness of the blood, which prevents any transudation, until the tension has much increased, and then causes the effusion of its fibrinous part, as well as of a much larger proportion of albumen. "I have been almost induced," says Dr. Williams, "to suppose that the polypous concretions and pseudo-membranous films occasionally effused on mucous surfaces, may result from long-continued congestion, with a highly fibrinous state of the blood. I have seen these evacuated from the air-tubes in one case, and in another from the intestines, from time to time for months, and even years, without symptoms of inflammation, but under circumstances rendering it probable that congestion was present. Extensive disease of the heart existed in one case, and amenorrhea in the other." (p. 149.) Dr. Williams states that he has been for several years in the habit of referring albuminous urine to congestion of the kidney,—a view, of which Mr. Robinson's experiments have given almost absolute demonstration. The following are the considerations which have led him to this opinion:

"1. The urine often becomes albuminous during great embarrassments of the circulation, in cases of organic disease of the heart, when the kidneys are otherwise healthy. 2. I have, in a few instances, observed temporarily albuminuria during the congestive stage of eruptive fevers. 3. In granular degeneration of the kidney, the amount of albumen in the urine is augmented by circumstances causing congestion of the kidney, and is reduced by remedies suited to remove this. 4. Bright's disease of the kidney, in its earliest stage, presents the appearance of a highly congested structure, and is excited by causes calculated to produce congestion; such as frequent irritation of the kidneys by stimulating liquors, congestion from exhausted tone, continued exposure to cold, especially after the kidneys have been much excited,—congestion from intropulsion: scarlatina probably operates as the two last combined. 5. The albumen in the urine abounds most in the congestive (first) stage of Bright's disease, the vessels becoming more or less obliterated in the progress of the disease, by a deposit of lymph in the cortical substance, and perhaps especially in the corpora Malpighiana,—which deposit is, at the same time, the cause which perpetuates some degree of congestion, whilst it supersedes the proper secreting structure." (p. 150.)

Dr. Williams speaks of "a variety of *hypertrophy*," as one of the results of continued congestion; but we think the term here misapplied. Hypertrophy consists in an increase of the *natural texture* of the part, which no mere *congestion* can produce; and the deposits which result from this state, if organized at all, are but very imperfectly so, and have no affinity with the surrounding textures, as Dr. Williams well points out in the following passage: "The hypertrophy resulting from congestion is probably not of a uniform kind, comprising equal growth of all the textures; but, arising from an effusion of lymph from the most congested vessels, it is an intervascular deposit,—at first mottling and exaggerating the appearance of the natural structure, as seen in the nutmeg liver and in the early soft stage of granular degeneration of the kidney, afterwards contracting and compressing the natural structure, and ultimately causing its condensation and atrophy, whilst the new deposit itself forms a granular or nodulated texture of low vitality. Such, I believe, to be the nature and origin of cirrhosis of the liver, and granular degeneration of the kidney." (p. 151.) Such deposits *may* have an indistinct fibrous structure, resembling that of areolar tissue; but this only when there is an unusual degree of plasticity in the blood,—a state bordering on inflammation, and passing into it.

We must pass over the valuable portion of this section, which treats of the remedies for congestion, without comment; but we shall extract the following passage, as affording important practical hints:

"The operation of several of the foregoing agents, in combination or succession, is generally more effectual than that of single ones, in the cure of congestions. Thus, congestion of the liver may resist the action of mercury, and may even be aggravated by it, until the vascular distension has been partially reduced by local bloodletting or derivants; then the mercury, by increasing the secretion, reduces the remaining congestion. Congestion of the kidneys is augmented rather than diminished by diuretics, which then fail to increase the secretion of urine, but may only render it more albuminous. But after some relief has been given by cupping to the loins, and hydragogue purgatives and diaphoretics, then some diuretics, particularly digitalis and cantharides, cause a freer flow of urine, with less albumen." (p. 154.)

In the succeeding section, on Determination of Blood, we meet with views, which appear to us equally imperfect with those on which we have just been commenting in regard to congestion. In our apprehension it is clear that, in a large proportion of cases, increased vital action of the part, whether that action be one of formation or of secretion, is the *cause*, not the consequence, of the increased afflux or determination of blood towards it. This would seem the almost necessary inference from the known effects of stimulants in occasioning such determination; and from the varying effects of the same stimulant on different parts, according to the degree of exalted action which it excites there. On our view of the causes affecting the circulation in the capillaries, an increased rate of motion, and an increased demand for blood, are the necessary consequences of this heightened function; and the enlargement of the arteries that supply the capillary system, so as to permit an increased flow of blood, will be a subsequent change. Dr. Williams, however, thinks differently. After noticing the cases in which determination of blood results from an increase of the *vis a tergo*, he continues, in regard to those more numerous cases in which no such increase exists:

"Is determination of the blood caused by *increased* action of the arteries? The only active property which we know these vessels to possess, is that of slow or tonic *contraction;* and such contraction of arteries leading to a part would diminish instead of increasing the motion and quantity of blood proceeding to the part. We may answer, from direct observation as well as from reasoning, that determination of blood is effected by enlargement of the arteries; and this enlargement is the effect of the arterial distension from behind, acting on a tube which has already lost some of its contractile power. The tonicity of the arteries makes them naturally resist the distending influence of the mass of blood pumped into them by the heart; but if this tonicity be impaired in any part, that of other parts forces the blood in augmented quantity into it, by which it is distended, and becomes an enlarged channel for the transmission of more blood and more force. If the arteries are enlarged, the capillaries and veins leading from them will be also enlarged, and will share the increase of blood and motion thus supplied to them. We find proof of the enlargement and distension of arteries leading to an inflamed or irritated part, in their increased and harder pulse; the coats of the vessels being stretched to tightness, the pulse is no longer softened by the usual elastic spring. So, too, in the frog's web gently irritated by an aromatic water, we see the arteries become enlarged, supplying a larger and more rapid flow of blood to the capillaries and veins, which all become enlarged also; and the whole vascular plexus, including vessels which before scarcely admitted red particles, then become the channels of a much increased current. This is determination of blood." (p. 157.)

In seeking for a physiological cause of the enlargement of the arteries in determination of blood, Dr. Williams justly observes that all that is known of animal physics opposes the idea of there being any power of "active dilatation" in the arteries; and he, whilst attributing it to a "weakening or reduction of the tone" of the arterial walls, is inclined to think that the nervous system is concerned, occasionally at least, in producing this, *blushing* being a "pregnant instance."

Now we are quite at a loss to know what is the distinction which he draws, between the efficient cause which produces congestion, and that which occasions determination; both conditions being regarded by him as dependent, as our readers will have perceived, on diminished tone, and consequent distension of the vessels of the part by the vis a tergo. How he explains the *diminished* motion in the one case, and the *increased* motion in the latter, we are totally unable to understand. These seem to us the essential elements of the two conditions, depending respectively upon the depression or exaltation of the vital functions of the part. In congestion, there is doubtless a diminished tonicity of the arteries; which, as we have seen, will increase the obstruction which may have begun in the capillaries, and will thus render the stagnation more complete, and the vascular tension greater. In determination of blood, on the other hand, there seems to us no evidence whatever, that the vital properties of the arteries are depressed; and from the causes which produce this state, no such effect could be anticipated. For all these causes are of such a nature, as to stimulate (for a time at least) the natural actions of a part; as may be seen in the determination of blood to the ovaries, uterus, and mammæ, at the periods of their functional excitement; or in the increased afflux of nutrient fluid to any set of muscles whose action has been peculiarly vigorous. We cannot but think that the *vis a fronte* thus generated is the truer explanation of the increased distension of the arteries leading to the part; but we would not shut out the influence of the nervous system, which may *permit* that distension in the manner suggested by Dr. Williams. This, indeed, was long ago suggested by ourselves. (Vol. VIII.) When the determination is long-continued, however, as is normally the case in regard to the uterus and mammæ during pregnancy and lactation, the arteries become enlarged by increased nutrition of their walls; a circumstance which strikingly distinguishes their state in a determination, from their condition in congestion. But there cannot be a more manifest proof of the opposite vital condition of the part itself, in these two states, than is afforded by its difference of functional condition. For whilst in congestion we have depressed function and deficient plastic action in the part--so that the plastic matter of the blood, not being drawn off by the normal process of nutrition, is effused in an unorganized or imperfectly organized form—there is, in determination of blood, exalted function of the part, accompanied by increased nutrition or true hypertrophy, if the determination should continue long enough, or should frequently recur. There is an exception, however, in regard to determination to the *head*, which may produce the opposite symptoms of excitement or depression; but this exception is explained by Dr. Williams, in accordance with the peculiar conditions of the cerebral circulation. He has noticed that "fits of epilepsy and convulsive hysteria are immediately preceded by throbbing of the carotids, which shows that the determination of blood is the proximate cause of the paroxysm. Drs. Darwin and Parry relate cases in which convulsive fits were prevented by pressure on one of the carotids; and I have practised this expedient with success in several cases. Many of the epileptic patients whom I have questioned have stated that the fit is always preceded by palpitation; which, for reasons before explained, sometimes peculiarly determines blood to the head." (p. 155.) The efficiency of a partial interruption of the current of blood going to the part, in lowering its action, is perfectly consistent with our view, that the origin of this state of exalted function is in the part itself; for if the *pabulum* to its operations be not supplied in sufficient quantity, these actions must necessarily be lowered.

We have now discharged our consciences, as to the critical examination of a part of Dr. Williams's most valuable treatise, in which it appears to us that he has been led astray, by his manifest indisposition to admit any other cause than the heart's action, as the moving force in the capillary circulation. We are far from wishing to assert dogmatically that he is wrong and that we are right; but we do feel that our doctrine, besides being the result of a more extended induction of physiological facts, enables us to give a more consistent view of the pathological phenomena of congestion and determination, than we find developed in his account of these subjects; and we shall elsewhere endeavour to show, that it more fully explains the complex phenomena of inflammation also.

The more pleasing task now remains, of briefly noticing the remaining portion of the work; which we are compelled, from the extent our review has already reached, to pass over with little but an enumeration of the topics it embraces, and a general indication of the manner in which these are treated.

The chapter which follows the discussion of inflammation and its results, treats of the Structural Diseases, or Diseases of Nutrition; which are the consequences of the various abnormal states of the blood, and of the solid textures, which have been previously considered. We think that Dr. Williams has done wisely in devoting a separate division of his treatise to this subject, although many portions of it might be considered to belong to the heads already passed over; for structural disease cannot, as he justly remarks, be always strictly distinguished into ultimate and proximate elements; and although we might infer from analogy that the several textures are singly affected in structural disease, yet we find the morbid alteration rarely confined to one anatomical element, but rather affecting an organ or part as a whole. It is not the object of Dr. Williams's treatise to give details of structural disease, which belong rather to the department of morbid anatomy; but he aims at tracing the chief forms of alteration in the nutritive processes, in which these diseases originate. It must be confessed that our knowledge on these subjects is as yet very limited; but Dr. Williams has made the most of what we possess; and we know no other account of these conditions which can be compared with his, either in richness of detail, (wherever the materials were within his reach,) philosophical treatment of general principles, or *suggestiveness* of character. The following table, exhibiting the classification which he adopts, will afford an outline view of his mode of treating the subject:

Elements of Structural Disease.

- *Diseased Nutrition*

  - Increased = Hypertrophy.

  - Diminished = Atrophy.

  - Perverted .

    - Induration.

    - Softening.

    - Transformation.

    - Deposits

      - Euplastic

        - Cicatrices.

        - False membranes.

      - Cacoplastic

        - Cirrhosis.

        - Fibro-cartilage.

        - Gray tubercle.

        - Atheroma, &c.

      - Aplastic

        - Yellow tubercle.

        - Calcareous matter, &c.

    - Growths

      - Non-malignant.

        - Cysts.

        - Tumours.

        - Hydatids, &c.

      - Malignant

        - Carcinoma.

        - Encephaloma.

        - Melanosis, &c.

- *Altered Mechanism*

  - Contraction.

  - Dilatation.

  - Obstruction.

  - Compression.

  - Displacement.

  - Rupture, &c.

If there is one part more than another which we would recommend to the special consideration of our readers, it is that which treats of Tubercle, and its connexion with constitutional states. We believe that Dr. Williams may justly take credit to himself for being the first promulgator of the doctrine, that tubercle is nearly related to the ordinary fibrinous and albuminous constituents of the blood, being a degenerated or caco-plastic form of the plasma, which ought to be the pabulum for the supply of the several tissues and organs; and that the tendency to its deposition should be considered, therefore, as a *diathesis*, to be treated by constitutional remedies, especially those which improve the processes of nutrition and assimilation. The caco-plastic nature of tubercle has been abundantly proved by the recent microscopic observations of Gerber, Addison, Gulliver, and others; which have shown that it may present various gradations of organization, from a texture consisting of imperfectly formed fibres and cells, to one which exhibits scarcely a trace of definite structure. There is a corresponding variety in the results of their deposition; the less degraded or degenerating forms of tubercle having a tendency to contract, and to produce atrophy of the organ; whilst the more degraded or aplastic pass on to the stage of softening and disintegration.

The concluding chapters embrace Nosology, Semeiology and Diagnosis, Prognosis, and Prophylaxis or Hygienics. The author avoids, and, we think, with wisdom, entering into any detail on the subject of nosology; but contents himself with indicating the principles, on which definitions and classifications of disease should be founded. The next subject, semeiology, is more fully and systematically treated. The indications of diseased action are divided by Dr. Williams into *physical signs* and *vital symptoms*, on the principles specified in the following extracts:

"*Physical signs* are those physical properties of the body, or of a part of it, which are perceptible to any of the senses of the observer. Thus, the form, size, colour, firmness or softness, weight, heat, and odour of the whole body, may be said to give physical signs or evidence of its condition, whether in health or in disease. So also, the form, size, colour, resistance, position, temperature, smell, and acoustic properties of a part of the body, afford physical signs of its condition, whether in health or disease. Thus the appearance of an external disease, the feeling of a solid tumour or of the fluctuation of liquid in the abdomen, listening to sounds produced by or in diseased internal parts, furnish us with physical signs of the presence of disease." (p. 356.)

"*Vital symptoms* are those phenomena which depend on *vital* properties of a part or parts of the body. Thus, irritability, tonicity, sensibility, excito-motion, secretion, and the more complex functions resulting from combinations of these elementary vital properties in a natural state, produce the symptoms of health; in an altered state, constitute the symptoms of disease. Hence vital symptoms have also been called *functional* symptoms, and *physiological;* but both these terms are objectionable, because both *function* and *physiology* relate likewise to physical properties, and would, therefore, include physical signs." (p. 359.)

Now we much doubt whether it is possible to make any division of the phenomena presented by disease, that shall be at the same time philosophical in character, and practically useful; and we cannot think that Dr. Williams has succeeded better than his predecessors. In fact, it seems to us that his usual acuteness has somewhat failed him in the analysis of this difficult subject. For do not the *physical signs* of disease depend, equally with the *vital symptoms*, "upon the vital properties of a part or parts of the body?" Does not an offensive odour, for example, result from some act of decomposition, occasioned by a disorder of the nutrient function? and is not the fluctuation of an abscess, and the resistance of a tumour, equally dependent upon morbid vital processes, with the pain which the patient experiences? Or, on the other hand, is not the disordered *motion* of a part—as, for example, the unequal elevation of the ribs on the two sides, or any form of convulsive action—as much a *physical* sign as its form, size, or colour? The least exceptionable mode of classifying morbid phenomena, as it appears to us, is that which regards the *signs* as comprehending *all* those of which the senses of the observer can inform him; and the *symptoms* as including those, for his information in regard to which he is necessarily dependent upon the statements of the patient. This division is of considerable practical utility in cases where we have reason to suspect a wilful or undesigned perversion of the truth, as in feigned disease or hysteria; but, in general, we doubt whether *any* classification can supersede the necessity of careful and discriminating analysis in each individual case. Notwithstanding our difference of opinion from Dr. Williams on the foregoing points, we strongly recommend this chapter to the perusal of our readers; as they will find in it many valuable hints, derived from extensive practical experience, as well as from sagacious reasoning.

The subject of diagnosis is passed over very cursorily; we could wish that it had been treated in a manner more conformable to its importance. The following general remarks are most true and valuable:

"Thus every department of medical knowledge is brought to bear on diagnosis; and in no branch is the information as well as the judgment of the practitioner more brought to a test. Natural shrewdness and tact, with some general knowledge of the nature and treatment of disease, may sometimes enable a comparatively ignorant person to practise medicine with an appearance of success; but such a person can make no hand of diagnosis; and he wisely either evades the whole subject, or expresses his opinion in vague terms, and scrupulously avoids their being brought to the test of the scalpel. The well-informed practitioner, on the other hand, feels that this is the subject which requires the full application of his mental powers and knowledge, as well as the keen exercise of his powers of observation; and in proportion as his senses are practised in observing, his information well arranged in relation to what he observes, and his judgment matured in discriminating and deciding the results, so will he be successful in forming his diagnosis, and in applying it to prognosis and practice." (p. 367.)

The chapter closes with this judicious and practical observation:

"The object of a complete investigation of the state of the patient is not merely to determine the particular disease under which the patient labours, but to discover what is healthy as well as what is morbid in his condition. The prognosis, or estimation of the amount and event of the disease, and the application of treatment, requires this full investigation. We have to consider, not merely *disease in the body*, but *the body in disease;* and it is by losing sight of this great practical axiom, that minute or microscopic inquirers, who may be singularly successful in special diagnosis, signally fail in prognosis and in practice." (p. 368.)

The subject of Prognosis is treated in the most general manner; and might, we think, have been more dwelt on with advantage; since there is nothing which the young practitioner requires, in reference to this subject, so much as *principles*, by the judicious application of which alone can he manifest any superiority to the empirical predictions made by ignorant persons who have been long habituated to the observation of disease. From this topic, Dr. Williams passes on to the consideration of the different *modes of death;* which naturally follows the inquiry into the relative importance of different symptoms. They are thus classified by him:

| | | |
|---|---|---|
| Death (cessation of function) beginning at the heart | { Sudden | = Syncope. |
| | { Gradual | = Asthenia. |
| ,, beginning at the breathing apparatus | | = Asphyxia, or apnea. |
| ,, beginning at the brain . | | = Coma. |
| ,, beginning at the medulla . | | = Paralysis. |
| ,, beginning in the blood . | | = Necremia, (νεκρος, dead; αιμα, blood.) |

Dr. Williams thinks it better to distinguish the mode of death by asphyxia, in which the *first* obstruction is in the breathing apparatus, from that which results from coma or paralysis; although the *immediate* cause of death is the same in both instances, viz. stagnation of blood in the pulmonary capillaries, and consequent interruption of the circulation. For practical purposes, this is certainly desirable; but we should have thought that the classification should have stood thus:

| | | |
|---|---|---|
| Death from interruption to the pulmonary circulation | { beginning in the breathing apparatus | = Apnea. |
| | { beginning in the nervous centres | = Coma and paralysis. |

We do not see that any distinction ought to be drawn between death beginning at the brain (coma), and death beginning at the medulla (paralysis); for coma need not prove fatal, unless the medulla oblongata be affected, in such a manner as to induce partial or complete suspension of the respiratory movements. This is clearly proved by the occasional persistence of hysteric coma—in which there is a complete suspension of the cerebral functions, without any affection of the medulla,—for days and even weeks, without the slightest tendency to asphyxia; and by the results of Flourens' experiments on the complete ablation of the cerebral hemispheres. Dr. Williams further points out the influence which the destruction of a considerable part of the spinal cord will possess on the functions less immediately connected with the maintenance of life, so as at last to cause the death of the whole system, by the suspension or alteration of a large number of its less important acts of nutrition, secretion, &c.: and he suggests the interesting inquiry, whether the gangrene of the lower extremities, sometimes induced by the use of ergotted corn, has not a connexion with injured function of the spinal cord. The following is his account of *Necremia:*

"Necremia, or death beginning with the blood, are terms which I venture to give to those fatal cases, in which the first and most remarkable change is exhibited by the blood. In typhoid fevers and others of the malignant or pestilential kind, none of the solids of the body constantly exhibit such an early change of function or structure, as would warrant us in tracing disease or death to them. It is true, that the functions of many solids are impaired,—the muscular and nervous systems, secretion, digestion, assimilation, and nutrition, all suffer; but the very universality of the affection seems itself to point to some cause more general than can be found in any individual function; and such a cause may be found in the blood. The blood, at an early period of these diseases, when they occur in their worst form, exhibits changes which show that disorder begins with it, and this disorder may reach to a fatal degree. The appearance of petechiæ and vibices on the external surface, the occurrence of more extensive hemorrhages in internal parts, the general fluidity of the blood, and frequently its unusually dark or otherwise altered aspect, its poisonous properties as exhibited in its deleterious operation on other animals, and its proneness to pass into decomposition, point out the blood as the first seat of the disorder, and by the failure of its natural properties and offices as the vivifier of all structure and function, it is plainly the medium by which death begins in the body. How far the change in the blood is in its structure and vital properties, or in its chemical composition, further research alone can determine; the vivifying function of the blood depends on all these combined, and it is this function which obviously fails. Hence the complete adynamia, or general prostration of all the living powers, which occurs where this source of death is most powerful. The blood, the natural source of life to the whole body, is itself dead, and spreads death instead of life. Almost simultaneously, the heart loses its power, the pulse becoming very weak, frequent, and unsteady; the vessels lose their tone, especially the capillaries of the most vascular organs, and congestions occur to a great amount; the brain becomes inactive, and stupor ensues; the medulla is torpid, and the powers of respiration and excretion are imperfect; voluntary motion is almost suspended; secretions fail; molecular nutrition ceases; and at a rate much more early than in other modes of death, *molecular* death following close on *somatic* death—that is, structures die and begin to run into decomposition, as soon as the pulse and breath have ceased; nay, a partial change of this kind may even precede the death of the whole body." (p. 386.)

We quite agree with Dr. Williams in this view of the subject, which we had ourselves been previously led to entertain by similar considerations; and we should be glad to follow him through his application of the principle here laid down to the pathology and treatment of the various endemic, epidemic, and infectious diseases, in which it operates to a greater or less extent. But this our limits forbid; and we shall refer our readers to the work itself, for this as for many other excellent examples of the connexion by sagacious reasoning—both inductive and deductive—

between sound principles on the one hand, and valuable practical observations on the other.

In another article in our present Number (art. IV, p. 344) will be found a series of facts, which tend to establish another mode of death, occurring in diseases of exhaustion, namely, general reduction of the temperature of the body, consequent upon exhaustion of its calorifying materials. It is well known that all the vital actions in the warm-blooded animals require, as one of their conditions, the maintenance of a temperature above the usual average of that of the atmosphere; and it necessarily follows, that if the heat of the entire body be permanently depressed, they must come to an end; and this, not in any one part alone, causing a cessation of a particular function, but in all the organs at the same time, giving a simultaneous check to their actions. We should be ourselves inclined, in considering the modes of death, to regard necremia and loss of heat as the two *most general* forms of dissolution; because they operate on the whole system alike and *simultaneously;* and then to consider the forms in which a single organ is primarily affected, as in syncope, and asphyxia, producing somatic death, on account of the general cessation of function, which *follows* the suspension of its own actions.

We are disappointed at finding under the last head Prophylaxis and Hygienics, nothing but an apology from the author, for being prevented, by want of time, from treating these subjects as he had intended. We hope, for the sake of the student and young practitioner, that this deficiency will be remedied hereafter; since we are convinced that there is no department of medicine which has been so much neglected, both in theory and practice; and to the increased cultivation of which we are to look for so much improvement in the application of science to the general welfare. One other deficiency we would indicate as capable, in our opinion, of being advantageously supplied in this treatise. It may be said that the subject of general therapeutics is introduced and discussed in almost every portion of the volume; but we should like to see the *principles* which should guide the administration of medicines, and which govern their action on the economy, brought together in as complete a form as the present state of our knowledge on the subject admits, even though the doing so might involve some repetition. We cannot think that, even with these additions, the work need be extended so much beyond its present very moderate limits, as to prevent it from becoming the text-book of every intelligent student and young practitioner. It is not to these alone, however, that we would recommend it; but to *all* who do not consider themselves too old to learn,—who, remembering the *ars longa, vita brevis,* are unwilling to regard their education as being completed in the years of their pupillage, but desire to avail themselves of the results of the extended inquiries now going on in the various departments of pathology, combined and generalized by an experienced teacher and skilful practitioner,—and who look for honorable distinction, either by their skill in the treatment of disease, or by the advances they may themselves make towards a further knowledge of its mysteries. To all such, then, we strongly recommend the perusal of Dr. Williams's 'Principles of Medicine;' in the confidence that, whether or not they see *all* the merit in it which we have stated it to possess, they cannot peruse it attentively and engraft its contents on their own minds without benefit to themselves, and consequently to their patients.

